# Effect of supplementation with vitamin D on biochemical markers of iron status and erythropoiesis in older people: BEST-D trial

**DOI:** 10.1017/S0007114525103516

**Published:** 2025-07-14

**Authors:** Abigail A. Lamikanra, Hoi Pat Tsang, Alireza Morovat, Harold Hin, Jonathan Emberson, Michael Hill, Robert Clarke, Jane Armitage, David J. Roberts

**Affiliations:** 1 NHS Blood and Transplant, Oxford, UK; 2 BRC- Haematology Theme and Nuffield Division of Clinical Laboratory Sciences, Radcliffe Department of Medicine, University of Oxford, Oxford, UK; 3 Department of Clinical Biochemistry, Oxford University Hospital Trust, Oxford, UK; 4 Hightown Surgery, Banbury, Oxfordshire, UK; 5 Clinical Trial Service Unit and Epidemiological Studies Unit, Nuffield Department of Population Health, University of Oxford, Oxford, UK; 6 Department of Haematology, John Radcliffe Hospital, Oxford, UK

**Keywords:** Randomised Controlled Trial, Vitamin D supplementation, Healthy older population, Iron, Erythropoiesis

## Abstract

Previous observational studies suggested that vitamin D may control the absorption of iron (Fe) by inhibition of hepcidin, but the causal relevance of these associations is uncertain. Using placebo-controlled randomisation, we assessed the effects of supplementation with vitamin D on biochemical markers of Fe status and erythropoiesis in community-dwelling older people living in the UK. The BEST-D trial, designed to establish the optimum dose of vitamin D3 for future trials, had 305 participants, aged 65 years or older, randomly allocated to 4000 IU vitamin D3 (*n* 102), 2000 IU vitamin D3 (*n* 102) or matching placebo (*n* 101). We estimated the effect of vitamin D allocation on plasma levels of hepcidin, soluble transferrin receptor (sTfR), ferritin, Fe, transferrin, saturated transferrin (TSAT%) and the sTfR–ferritin index. Despite increases in 25-hydroxy-vitamin D, neither dose had significant effects on biochemical markers of Fe status or erythropoiesis. Geometric mean concentrations were similar in vitamin D3 arms *v*. placebo for hepcidin (20·7 [se 0·90] *v*. 20·5 [1·21] ng/ml), sTfR (0·69 [0·010] *v*. 0·70 [0·015] µg/ml), ferritin (97·1 [2·81] *v*. 97·8 [4·10] µg/l) and sTfR–ferritin ratio (0·36 [0·006] *v*. 0·36 [0·009]), respectively, while arithmetic mean levels were similar for Fe (16·7 [0·38] *v*. 17·3 [0·54] µmol/l), transferrin (2·56 [0·014] *v*. 2·60 [0·021] g/dl) and TSAT% (26·5 [0·60] *v*. 27·5 [0·85]). The proportions of participants with ferritin < 15 µg/l and TSAT < 16 % were unaltered by vitamin D3 suggesting that 12 months of daily supplementation with moderately high doses of vitamin D3 are unlikely to alter the Fe status of older adults.

The most common nutritional cause of anaemia in developed and developing countries is Fe deficiency affecting about one-third of the global population^(1)^. Fe deficiency occurs following insufficient intake of Fe to meet the physiological needs (growth or pregnancy) or pathological loss of Fe or excessive blood donation^(2)^. Fe deficiency without anaemia can exacerbate physiological impairment in vulnerable groups including the elderly which adds to the public health burden of age-related disease worldwide^(3)^.

While many diets are low in Fe, sub-maximal absorption of dietary Fe and Fe supplements may contribute to Fe deficiency^(2)^. Fe absorption and mobilisation of Fe stores are enhanced in Fe deficiency when plasma levels of the peptide hormone hepcidin are low where enhanced levels of hepcidin prevent expression of the Fe transporter ferroportin so that Fe is not mobilised from the small intestine or macrophages^(4,5)^. Against the background of diurnal variation^(6,7)^, hepcidin is increased not only by high Fe stores but also in response to inflammation in chronic disease^(8,9)^. Thus, in older populations where inflammation and chronic disease exist, there is reduced absorption and reduced bioavailability of Fe and subsequent exacerbation of co-morbidities^(10,11)^.

We wanted to investigate whether increasing Fe availability through modulation of hepcidin levels could be an alternative to administering oral or parental Fe. Vitamin D has been linked with multiple cellular functions that control the absorption of Fe^(12)^ and an experimental study demonstrated that the active form of vitamin D can reduce hepcidin (HAMP) gene expression and HAMP mRNA levels in hepatocytes^(13)^. However, the evidence to support vitamin D supplementation with effective doses of 50 µg or more is limited to small, randomised clinical trials (RCT) with less than fifty participants and population-based observational studies, in healthy young adults with mean ages between 28 and 40 years^(14–16)^.

The most direct indicator of Fe stores is ferritin. However, plasma levels of ferritin are increased in response to infection and inflammation^(17)^. In isolated Fe deficiency, the Fe saturation of transferrin (TSAT%) is reduced to less than 16 %^(18)^ and an increase in soluble transferrin receptor (sTfR) above normal concentrations (e.g.1·55 µg/ml) is observed for Fe-deficient erythropoiesis^(19)^. Since ferritin levels may be altered in the setting of low-grade inflammation, independently of effects on Fe stores, measuring plasma levels of transferrin saturation, sTfR and hepcidin and ferritin should provide a more reliable and comprehensive assessment of Fe status, Fe bioavailability and erythropoiesis in population studies. Few studies have examined the relationship between vitamin D supplementation and Fe status using these markers of Fe status in older adults.

Therefore, the aim of this study was to assess if dietary supplementation with moderate or high daily doses of vitamin D3 affects biochemical markers of Fe status or erythropoiesis in healthy older adults living in the UK. If vitamin D could alter absorption of Fe, then administration of vitamin D3 supplements or mandatory food fortification with vitamin D3 could provide a cost-effective and safe method to prevent Fe deficiency anaemia, avoiding problems associated with intestinal Fe absorption or expense of parental administration, in high-risk populations including infants, young women and blood donors in addition to older people.

We performed post hoc secondary analysis of a parallel group RCT in the BEST-D study, where a total of 305 community-dwelling men and women aged over 65 years in the UK were randomly allocated to receive one daily soft gel capsule containing vitamin D_3_ (4000 IU or 2000 IU equivalent to 100 µg or 50 µg daily) or matching placebo capsule for 12 months^(20)^. Using a double-blind study design, blood levels of 25-hydroxy-vitamin D (25[OH]D) and biochemical markers of Fe status, including hepcidin, ferritin, sTfR, TSAT% and Fe, were measured before and after 12 months of treatment in all participants.

## Methods

### Study design and interventions

The design and results of the BEST-D trial have been previously reported^(20–22)^ but selected details are provided in the online Supplementary Information. The BEST-D trial was a double-blind randomised placebo-controlled parallel group trial conducted to comply with the guidelines of the Declaration of Helsinki. The trial (ISRCTN07034656) was registered prospectively at https://doi.org/10.1186/ISRCTN07034656 and received approval from the National Research Ethics Service (NRES) Committee South Central–Oxford B (REC reference: 12/SC/0243), the Thames Valley Primary Care Research Partnership, a Clinical Trial Authorisation from Medicines and Healthcare products Regulatory Agency (MHRA) and is in the National Institute for Health Research (NIHR) Trial portfolio. Randomisation treatments used minimisation to ensure the balance of age and sex in each allocated treatment group. Participants were eligible if they were ambulant community-dwelling men and women aged 65 years and older, were from a single practice, were not nursing home residents and were not taking more than 400 IU (10µg) of vitamin D3 nor prescribed calcium supplements, bisphosphonates, parathyroid hormone or calcitonin. Participants that had medically diagnosed dementia or a history of hypercalcaemia, hyperparathyroidism, lymphoma, sarcoidosis, active tuberculosis or renal calculus; were judged by their doctor as likely to be poorly compliant with clinic visits or medication or had a history of alcohol or substance misuse or a history that might limit their ability to take the study treatment (e.g. terminal illness) were excluded. All participants provided written informed consent. Vitamin D3 or a matching placebo were administered as soft gel capsules provided by Tishcon Corporation. Volunteers were randomly allocated to take 4000 IU vitamin D3 (*n* 102), 2000 IU vitamin D3 (*n* 102) or a matching placebo (*n* 101) daily and were followed up for one year. All clinical, laboratory and statistical scientists were blinded to treatment allocation until all clinical and laboratory data collections were completed.

### Biochemical markers of iron status and erythropoiesis

In keeping with the primary aims of this study, plasma samples were measured at baseline prior to the onset of treatment and after 12 months of treatment. Plasma levels of 25(OH)D were measured using an Access 2 immunoassay analyzer (Beckman Coulter Ltd.) at the Wolfson laboratory, Nuffield Department of Population Health, Oxford. Hepcidin, sTfR (µg/ml), transferrin (Tf, g/dl), Fe (ng/ml) and ferritin (µg/l) were measured at the John Radcliffe Hospital, Oxford. Commercially available ELISA were used to measure bioactive hepcidin (DRG product EIA5782, Lot 314K038) and sTfR (Biovendor product RD194011100, Lot E17046). All measurements by ELISA were completed within 1 month of each other. The inter-assay CV% of the controls for these assays were 6·26–11·28 % and 5·25–5·52 %, respectively. Plasma levels of Fe were measured on an Abbott Architect c16000 automated analyser using a colourimetric method employing Ferene-S as chromogen. Transferrin was measured using an immunoturbidimetric assay on the same analyser. Ferritin was measured by a chemiluminescence microparticle two-site immunoassay on an Abbott i2000 auto-analyser. The quality of these assays was controlled by the use of three quality control specimens (low, mid and high) assayed at 4-hourly intervals, and then assessing results based on the mean and sd of TSAT% were determined from plasma concentrations of Fe × 100/ total Fe binding capacity (TIBC) where TIBC = [Tf] × 25·16^(23)^. The sTfR–ferritin index was calculated by dividing the sTfR value by the logarithm of the ferritin level and was used because it remains unaffected by inflammation, providing a clearer picture of Fe status in patients with chronic diseases or inflammatory conditions. The positive association of ferritin levels with hepcidin, Fe and TSAT% or non-positive associations with transferrin and soluble transferrin indicate that the technical measurements were consistent with expected findings for these parameters (see online Supplementary Figure 1 and Supplementary Table 3).

We used the WHO guidelines to define probable Fe deficiency as ferritin < 15µg/l^(24)^ or transferrin saturation as < 16 %^(18)^ and < 30µg/l ferritin was explored as a threshold to lengthen the interval between each blood donation^(25)^. Other biochemical and physical measurements were previously carried out at baseline, 1 month, 6 months and 12 months as described previously^(20)^ and summarised in Table S1 of online Supplementary Information.

### Statistical analysis

All efficacy and safety assessments were conducted using an intention-to-treat principle and followed a pre-specified analytical plan for the BEST-D study as previously described^(20,21)^.

The primary objectives of this trial were to compare the effects on blood concentrations of 25(OH)D and the proportion of participants with 25(OH)D concentrations > 90 nmol/l after 1 year of daily supplementation with 100µg (4000 IU) or 50µg (2000 IU) vitamin D3 *v*. placebo. The secondary outcomes included mean plasma 25(OH)D levels and percentage of participants with 25(OH)D > 90 nmol/l (36 ng/ml) at 1 and 6 months; percentage of participants with parathyroid hormone in the normal range (1·1–6·8 pmol/l) at 1, 6 and 12 months; percentage of participants with calcium levels above the normal range (2·15–2·55 mmol/l) at 1, 6 and 12 months and mean level at 6 and 12 months of biomarkers for cardiovascular risk factors as described in online Supplementary Information.

The sample size was selected to detect the smallest difference in mean 25(OH) concentrations at 12 months between the two doses. With 100 participants in each group and an assumed sd of 20 nmol/l, this study had 90 % power (at 2p = 0·01) to detect a true difference in mean 25(OH)D between the two active doses at 12 months of 11 nmol/ (previously described^(21)^). Although the BEST-D trial was not powered for the outcomes in these secondary analyses, the comparison of 204 participants allocated vitamin D *v*. 101 allocated placebo gives 80 % power at 2p = 0·05 to detect a 0·34 sd difference in each Fe biomarker (and 90 % power at 2p = 0·05 to detect a 0·4 sd difference).

In accordance with the BEST-D statistical analysis plan and pre-specified approach for the analysis of secondary outcomes, the two active doses were combined in the main analysis presented here, with all three arms only shown as online Supplementary information. The achieved differences in plasma levels of 25(OH)D at 6 and 12 months between those allocated 4000 IU *v*. 2000 IU daily and *v*. placebo were broadly similar when subdivided by the quartiles of plasma 25(OH)D levels at baseline^(20)^ and online Supplementary Table S2. Therefore, in this study, the effect of either 2000 or 4000 IU of vitamin D3 on Fe status at 12 months was compared to placebo as described previously^(21)^. Comparisons of mean follow-up values between treatment arms involved analysis of covariance adjusted, where possible, for the baseline value (with multiple imputations used to impute the few missing data). As is standard practice when presenting trial results, sd were used to describe the population, and SE were used when testing the difference between two means. To accommodate the presence of outliers, variables with skewed data (i.e. Ferritin, Hepcidin and sTfR) were presented as median and IQR and analyses done on the log scale before being transformed back to their original scale and presented as geometric means (with approximate SE). All p values were two-sided and considered statistically significant, without allowance for multiple testing, if they were < 0·05. Analyses were conducted using SAS version 9.3 and R version 2.11.1. Fisher’s exact test was used to compare the proportions of participants with low ferritin (< 15µg/l or < 30 µg/l) or TSAT% < 16 %. Box and whisker plots and simple linear regression lines were plotted using GraphPad Prism v10.1.

## Results

### Baseline characteristics of participants

The characteristics of male and female participants that were relevant to Fe status measurements are shown in Table 1 and online Supplementary Table S1. Overall, 51 % were men and 49 % were women, the mean age of all participants was 72 years. The mean BMI was 28 kg/m^2^ (24 % with BMI > 30^(20)^) mean levels of systolic and diastolic blood pressures were 131 mmHg and 77 mmHg, respectively. The mean estimated glomerular filtration rate was 77ml/min/1·73m^2^ (Table 1). With numbers balanced by treatment allocation groups, the proportion of participants with signs of underlying inflammation (i.e. CRP > 5mg/l) was 20 %; those with prior history of diabetes, heart disease, hypertension and diabetes were 9 %, 15 %, 39 %, respectively, and the proportion that reported joint aches and pains was 64 % (online Supplementary Table S1)^(20)^ suggesting the presence of low-grade inflammation in this otherwise healthy population. Compliance with the allocated treatment groups was reported as being consistently between 85 % to 92 %, and 94 % of participants attended their scheduled visits^(20)^.

**Table 1.** Baseline characteristics by randomised treatment allocation (Percentages; mean values and standard deviations; median values and interquartile ranges)

	4000 IU/day (*n* 102)			2000 IU/day (*n* 102)			Placebo (*n* 101)		
	%	Mean	sd	%	Mean	sd	%	Mean	sd
Age, years		71	6		72	6		72	6
Male	51 %			50 %			51 %		
Physical measurements									
BMI, kg/m^2^		27	5		27	4		28	5
Systolic blood pressure, mm Hg		133	21		132	17		129	19
Diastolic blood pressure, mm Hg		78	11		77	10		77	12
Estimated glomerular filtration rate, ml/min/1·73m²		77	11		77	10		76	12
25(OH)D, nmol/l		49	15		55	23		47	15
		Median	IQR		Median	IQR		Median	IQR
C-reactive protein, mg/l		1·54	0·71–3·05		1·87	0·78–4·03		1·75	0·71–4·32
Measures of Fe status									
Hepcidin, ng/ml		25·3	16·6–35·7		27·2	15·2–43·0		28·4	18·6–38·9
sTfR, µg/ml		0·65	0·58–0·78		0·68	0·60–0·83		0·68	0·60–0·77
Ferritin, µg/l		112·6	61·3–204·5		110·1	63·1–173·4		101·6	64·9–187·9
Ferritin < 15 µg/l	3 %			3 %			7 %		
Ferritin < 30 µg/l	10 %			13 %			13 %		
sTfR:ferritin index		0·33	0·27–0·42		0·34	0·28–0·46		0·35	0·29–0·43
		Mean	sd		Mean	sd		Mean	sd
Fe, µmol/l		17·2	4·7		15·8	5·7		15·9	5·3
Transferrin, g/dl		2·6	0·4		2·6	0·4		2·5	0·4
TSAT, %		27·4	9·0		24·7	9·6		26·2	10·3
TSAT < 16 %	8 %				20 %			17 %	

Values shown are means (sd) or %. Except for values for Fe biomarkers hepcidin, sTfR, ferritin and sTfR-Ferritin index that are skewed and so presented as median (IQR).

### Biochemical markers of iron status at baseline

**Figure 1.**
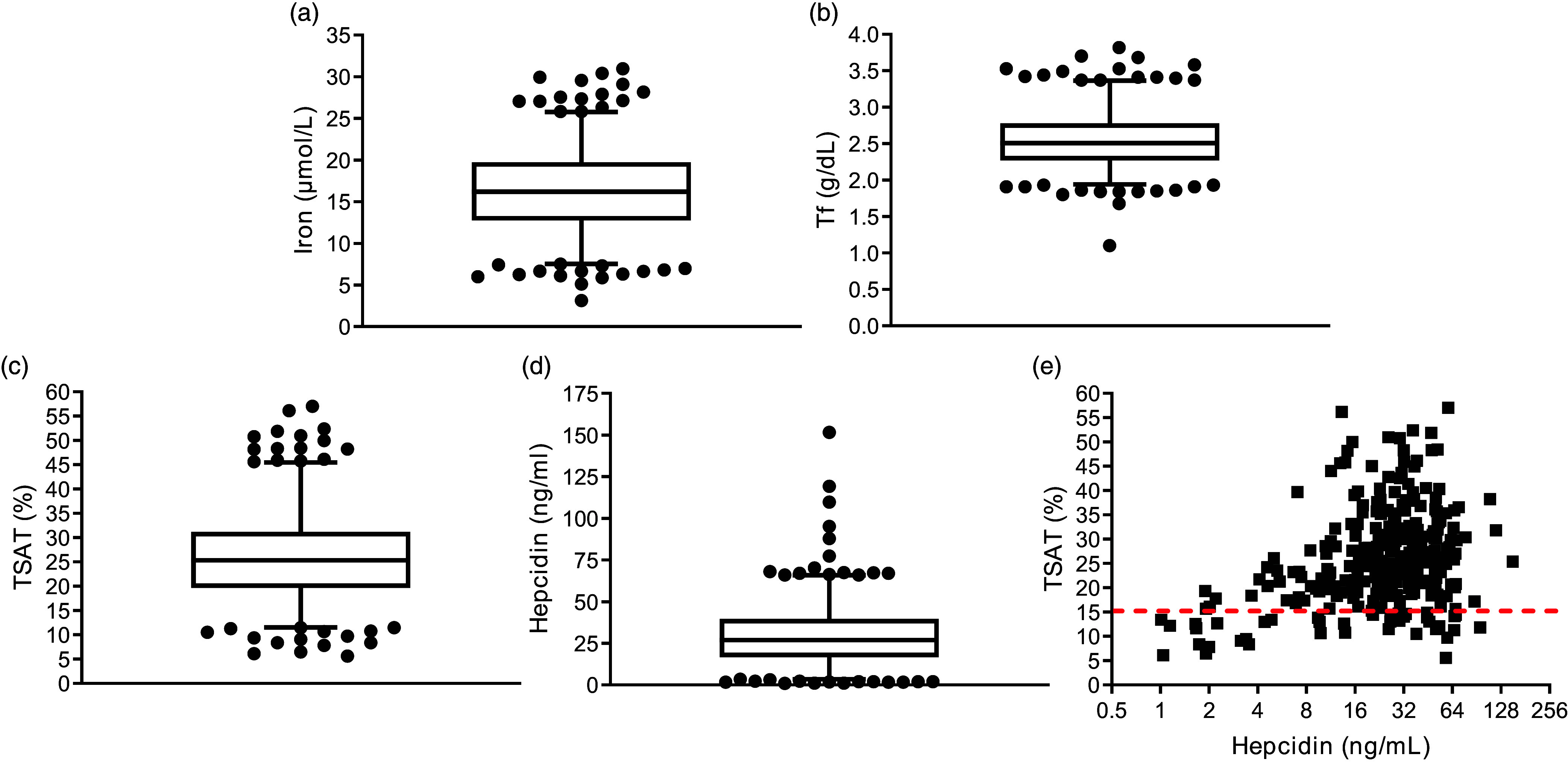
Median levels of parameters measured at baseline to define Fe deficiency in healthy male and female participants in the BEST-D study. Box and whisker plots show the median of each parameter with individual outliers shown above and below the inter-quintile range for (a) Fe, (b) transferrin (Tf), (c) transferrin saturation (TSAT%), (d) Hepcidin and (e) correlation of hepcidin with TSAT% (below dashed line may be deficient in Fe).

Blood biomarkers of Fe stores included serum ferritin and markers of Fe metabolism included serum Fe, transferrin, sTfR and hepcidin- and the derived TSAT%. Online Supplementary Figure 1 shows the distribution of these measurements. Approximately 15 % of the trial population were Fe deficient based on TSAT < 16 %, and 4 % deficient based on ferritin < 15 µg/l (Table 1). About half of those with low TSAT% did not have the correspondingly lower levels of hepcidin for this population (i.e., hepcidin < 8 ng/ml) (Figure 1(e)) and a small proportion (2·19 %) of the trial population had Fe saturation > 50 % (Figure 1(e)). Consistent with successful randomisation procedures, mean levels of these parameters were well matched between those allocated to either dose of vitamin D or placebo (Table 1).

### Biochemical markers of iron-deficient erythropoiesis

**Figure 2.**
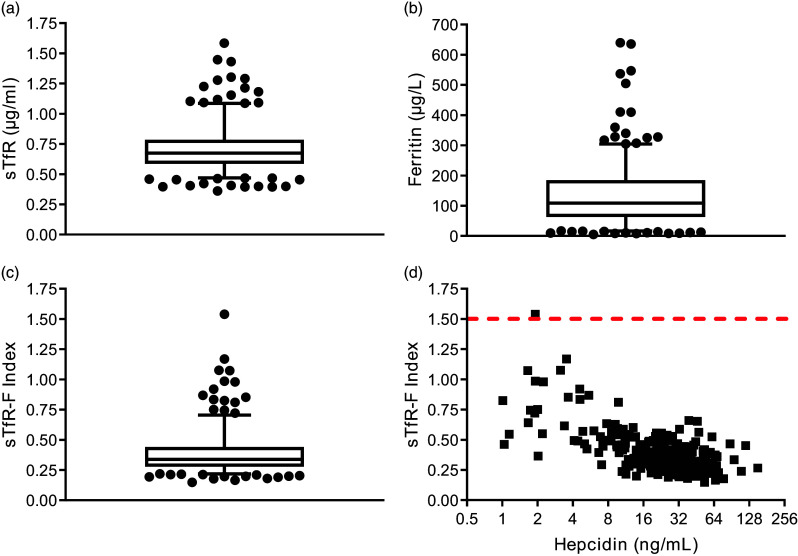
Measurements of Fe availability for erythropoiesis at baseline and their relationship with hepcidin levels in the BEST-D cohort of healthy male and female participants. (a) sTfR, (b) ferritin, (c) sTfR-ferritin index (sTfR-F index) is shown at baseline before randomisation. Horizontal bars of box and whisker plots represent medians with individual outliers above and below the inter-quintile range. (d) Correlation of hepcidin with sTfR-F index (above dashed line may have reduced erythropoiesis).

A sTfR > 1·5–2µg/ml or sTfR-Ferritin index > 1·5 is suggestive of Fe deficiency anaemia due to inadequate Fe for erythropoiesis in the bone marrow^(19,26)^. The median (inter-quintile range, IQR) sTfR was 0·68 µg/ml (0·6–0·8) and median (IQR) of sTfR-Ferritin was 0·34 (0·3–0·4) suggesting that most participants had sufficient red cell output from the bone marrow at randomisation (Figure 2).

### Effects of vitamin D on biochemical markers of iron status and erythropoiesis

Mean (sd) plasma 25[OH]D levels were about 50 (18) nmol/l at baseline and increased to 137 (39), 102 (25) and 53 (16) nmol/l for participants allocated to daily doses of 4000 IU (100 µg), 2000 IU (50 µg) and placebo, respectively. Overall, after 12 months of treatment among those allocated 4000 IU, 2000 IU or placebo, respectively, 88 %, 70 % and 1 % achieved a 25(OH)D levels > 90 nmol/l. Following 12 months of daily vitamin D3 at either dose of 2000 or 4000 IU, there were no significant differences in any of the biomarkers of Fe status or erythropoiesis. Geometric mean levels by allocation to either dose of vitamin D3 or placebo at 12 months were similar for hepcidin (20·7 [se 0·90] *v*. 20·5 [1·21] ng/ml), sTfR (0·69 [0·010] *v*. 0·70 [0·015] µg/ml), ferritin (97·1 [2·81] *v*. 97·8 [4·10] µg/l) and sTFR–ferritin ratio (0·36 [0·006] *v*. 0·36 [0·009]), respectively, while arithmetic mean levels were similar for Fe (16·7 [0·38] *v*. 17·3 [0·54] µmol/l), transferrin (2·56 [0·014] *v*. 2·60 [0·021] g/dl) and TSAT% (26·5 [0·60] *v*. 27·5 [0·85]) (Table 2). Furthermore, there was no evidence of effect on any markers of Fe status when stratified by baseline levels of 25(OH)D (online Supplementary Tables 4a and b)

**Table 2.** Effect of allocation to vitamin D3 4000 IU or 2000 IU daily *v*. placebo on 12-month concentrations of 25(OH)D and on markers of Fe deficiency anaemia (Numbers and percentages; mean values with their standard errors)

	Either dose (*n* 204)		Placebo (*n* 101)		*P*
	Mean	se	Mean	se	
25(OH)D, nmol/l	119·3	1·73	53·4	2·41	<0·0001
Hepcidin, ng/ml	20·7	0·90	20·5	1·21	0·89
sTfR, µg/ml	0·69	0·010	0·70	0·015	0·70
Ferritin, µg/l	97·1	2·81	97·8	4·10	0·88
	*n*	%	*n*	%	
Ferritin < 15 µg/l	5	2·5 %	6	6·0 %	0·19
Ferritin < 30 µg/l	19	9·3 %	9	8·9 %	1·00
	Mean	se	Mean	se	
Fe, µmol/l	16·7	0·38	17·3	0·54	0·39
Transferrin, g/dl	2·56	0·014	2·60	0·021	0·13
TSAT, %	26·5	0·60	27·5	0·85	0·36
	*n*	%	*n*	%	
TSAT < 16 %	27	13·2 %	10	9·9 %	0·46
	Mean	se	Mean	se	
sTfR–ferritin index	0·36	0·006	0·36	0·009	0·98

Mean (se) or *n* (%) is shown. Hepcidin, sTfR, ferritin and the sTfR–ferritin index were analysed on the log scale; their values correspond to the geometric mean and its approximate standard error. Estimates of mean and SE were adjusted for the baseline values, with missing data imputed using multiple imputations. Fisher’s exact test was used to compare proportions with low ferritin or TSAT.

## Discussion

The main findings of the present report of the BEST-D trial demonstrated that allocation to either 2000 IU daily or 4000 IU vitamin D3 daily for 12 months was associated with a 2-fold increase in plasma levels of 25(OH)D from 53·4 nmol/l at baseline to 119·3 nmol/l at 12 months, but this had no significant effect on any of the markers of Fe status or Fe deficiency in an older ambulatory group of men and women. Furthermore, the availability of Fe in participants who were deficient in vitamin D at the start of the study was unaltered after daily supplementation doses with 2000 IU (50 µg) or 4000 IU (100 µg) vitamin D3 for 12 months. Consequently, the present study provides reliable evidence that increasing vitamin D status in community-dwelling healthy older people with low levels of inflammation did not alter Fe status or Fe metabolism.

The results are consistent with the findings of a previous randomised trial of vitamin D3 in vitamin D deficient adults that reported that allocation to either 25 µg or 10 µg of vitamin D3 daily for 16 weeks did not alter blood levels of ferritin, haemoglobin, Fe or transferrin saturation^(27)^. In this trial, the mean (sd) age of participants was 38 (8) years and baseline 25(OH)D increased 2-fold to 49 nmol/l. Conversely, a small study of 28 healthy adults who were allocated to receive a single large dose of 250 000 IU vitamin D3 as a bolus *v*. placebo reported a 73 % reduction in hepcidin after 7 days although an increase of ferritin was absent^(15)^. Again, most participants were vitamin D deficient (25[OH]D < 20 ng/ml) at baseline and their mean (sd) age was 27 (6) years.

Overall, when these studies are considered together with the findings of the BEST-D trial, they suggest that the dose and timing of vitamin D administration, the prevalence of vitamin D deficiency, age of study participants and scheduling of hepcidin measurements after supplementation with vitamin D should be considered in the trial design when determining the effects of vitamin D on blood levels of hepcidin and other markers of Fe status in healthy adults.

RCT investigating the effects of vitamin D3 on erythropoiesis in patients with chronic kidney disease is difficult to interpret and differs from trials conducted in apparently healthy adults^(28,29)^. While these studies demonstrate that the dose and timing of measurements of hepcidin are critical, there is a high risk of inflammation in this patient population and, hence, a greater use of erythropoietin stimulating agents that may mask effects of vitamin D3 on hepcidin and other biochemical markers of Fe status.

Despite its robust design and effectiveness at increasing plasma levels of 25(OH)D, at baseline, most participants in BEST-D were not deficient in vitamin D (i.e. 25(OH)D < 30 nmol/l) so that any influence of vitamin D3 on hepcidin may be difficult to assess (online Supplementary Table 4b). We also did not record the use of Fe supplementation which could mask any effect of vitamin D3 on hepcidin or other markers of Fe status.

The statistical analysis plan for the BEST-D trial did not include prespecified post hoc analyses based on indicators of inflammation such as CRP > 5mg/l^(30)^. However, using the prespecified approach for analysing clinical trials, we saw no significant changes in TSAT% or sTfR-Ferritin indices to suggest that inflammation masked changes in Fe status after 1 year of supplementation with vitamin D. To avoid false positives due to multiple testing errors, our secondary analysis was limited to only include post hoc analysis of participant numbers with low Fe stores (ferritin and TSAT%) and assess the effects of vitamin D supplementation on the Fe status of participants that had insufficient levels of 25 (OH)D at baseline. We followed the primary aim of the BEST-D trial by assessing Fe indices at 12 months^(21)^ as it was beyond the scope of this secondary analyses to use earlier time points of 1 and 6 months. Hepcidin induced by inflammation in 25-year-old volunteers infected with *Salmonella* Typhi^(31)^ subsides within 48 h, whereas hepcidin levels in premenopausal women are reduced after 14 d treatment with ibuprofen^(32)^. We therefore cannot rule out that plasma hepcidin levels dropped at time points that were different to those specified for the BEST-D trial.

To determine if vitamin D3 increases the availability of Fe by reducing plasma levels of hepcidin, future RCT should be designed to assess the effects of treatment in populations deficient in Fe and vitamin D; stratify by sex and by inflammatory status and plan to monitor acute changes in hepcidin and other indices for Fe deficiency anaemia at the same and later time points. However, in the present trial of 305 older men and women with low levels of inflammation, administration of daily doses of vitamin D3 at 2000 or 4000 IU per day for 1 year did not alter any biochemical markers of Fe absorption or Fe stores or erythropoiesis.

## Supporting information

Lamikanra et al. supplementary material Lamikanra et al. supplementary material

## Supplementary material

For supplementary material/s referred to in this article, please visit https://doi.org/10.1017/S0007114525103516
