# Supplementary material for: Effect of supplementation with vitamin D on biochemical markers of iron status and erythropoiesis in older people: BEST-D trial
Source: Br J Nutr. 2025 Jun 2;134(1):28–34. doi: 10.1017/S0007114525103516 (PMC12379118; doi:10.1017/S0007114525103516)
Supplement: Lamikanra et al. supplementary material [file S0007114525103516sup001.pdf]

Supplementary Information

Table of Contents

Appendix. Primary and secondary outcomes of the BEST-D study 2

Table S1. Additional baseline characteristics by allocated treatment... 3

Table S2. Effect of allocation to 4000 versus 2000 IU daily vitamin D on plasma 25(OH)D at 6 and 12 months, by baseline 25(OH)D. 4

Figure S1: The association between iron stores in the form of ferritin with biochemical markers for iron release (hepcidin), transferrin (Tf), Tf saturation (TSAT) and red cell output from the bone marrow (sTfR and sTfR-ferritin index). 5

Table S3: Correlations between baseline measures of iron status.. 6

Table S4a: Post-hoc assessment of the effect of allocation to vitamin D3 4000 IU or 2000 IU daily versus placebo on 12-month concentrations of iron biomarkers subdivided by baseline 25(OH)D concentration (below/above median). 7

Table S4b: Post-hoc assessment of the effect of allocation to vitamin D3 4000 IU or 2000 IU daily versus placebo on 12-month concentrations of iron biomarkers subdivided by baseline 25(OH)D concentration (below/above 30 nmol/L)... 8

Appendix. Primary and secondary outcomes of the BEST-D study**.** Described in Supporting information for Hin et al 2017[20] and by Clarke et al 2015[21] .

The co-primary outcomes were mean plasma 25(OH)D levels and percentage of participants with 25(OH)D levels >90 nmol/L at 12 months.

Secondary outcomes included: mean plasma 25(OH)D levels, and percentage of participants with 25(OH)D >90 nmol/L (36 ng/mL), at 1 and 6 months; percentage of participants with PTH in the normal range (1.1-6.8 pmol/L) at 1, 6 and 12 months; percentage of participants with albumin-corrected calcium levels above the normal range (2.15- 2.55 mmol/L) at 1, 6 and 12 months; mean level at 6 and 12 months of albumin, phosphate, creatinine, alkaline phosphatase and lipids; and blood pressure recorded at 6 and 12 months.

Additional secondary outcomes included heart rate, blood pressure and brachial and digital arterial stiffness in all participants at 6 and 12 months.

Tertiary outcomes assessed at 12 months included all site and specific fractures, falls, muscle pain, joint pain, self-assessed physical activity, number of respiratory infections, geriatric depression score, weight, height, BMI, hand grip strength, physical performance measures, and bone density T- and Z-scores at the hand and wrist.

Safety outcomes included all serious adverse events, irrespective of whether these were related to study treatment, reasons for stopping study treatment and biochemical safety data.

Table S1. Additional baseline characteristics by allocated treatment. Taken from Hin, 2017[20].

	4000 IU/day (n=102)	2000 IU/day (n=102)	Placebo (n=101)
Age (years)	71 (6)	72 (6)	72 (6)
Female	50 (49%)	51 (50%)	50 (49%)
Male	52 (51%)	51 (50%)	52 (51%)
Current smoker	7 (7%)	7 (7%)	7 (7%)
Dietary calcium (mg/day)	724 (287)	695 (292)	713 (302)
Prior disease			
Heart disease *	20 (20%)	11 (11%)	11 (11%)
Stroke/TIA	5 (5%)	8 (8%)	6 (6%)
Hypertension	40 (39%)	44 (43%)	35 (35%)
Diabetes	9 (9%)	9 (9%)	9 (%)
Fracture (ever)	31 (30%)	30 (29%)	30 (30%)
Any fall in past 6 months	13 (13%)	15 (15%)	12 (12%)
Medication			
Any antihypertensive	50 (49%)	52 (51%)	46 (46%)
Statin	32 (31%)	29 (28%)	23 (23%)
Any antithrombotic	20 (20%)	23 (23%)	18 (18%)
Vitamin D (≤400 IU/day)	12 (12%)	10 (10%)	13 (13%)
Calcium	4 (4%)	1 (1%)	4 (4%)
Physical measurements			
Height (cm)	168 (10)	168 (10)	167 (10)
Weight (kg)	77 (17)	78 (15)	79 (15)
Body mass index (kg/m2)	27 (5)	27 (4)	28 (5)
Grip strength (kg)	25 (11)	25 (11)	25 (11)
Blood pressure and arterial stiffness			
Systolic blood pressure (mmHg)	133 (21)	132 (17)	129 (19)
Diastolic blood pressure (mmHg	78 (11)	77 (10)	77 (12)
Heart rate (beats/min)	66 (10)	66 (12)	65 (9)
Pulse wave velocity (m/s)	10.0 (1.9)	9.6 (1.6)	9.7 (1.8)
Aortic augmentation index (%)	38 (16	37 (14)	36 (15)
Pulse trace stiffness index (%)	9.2 (2.3)	9.1 (2.4)	9.5 (2.8)
Pulse trace reflection index (%)	64 (14)	63 (15	67 (12)
Physical activity and muscle/joint pain			
Physical activity rating (1-10)	6.5 (2.0)	6.2 (2.0)	6.5 (2.0)
Any muscle aches/pains	43 (43%)	35 (34%)	38 (38%)
Any joint aches /pains	66 (65%)	66 (65%)	64 (63%)

Mean (SD) or % shown. *Defined as heart attack, angina or heart failure

Table S2. Effect of allocation to 4000 versus 2000 IU daily vitamin D on plasma 25(OH)D at 6 and 12 months, by baseline 25(OH)D. Groups were defined retrospectively to summarize changes in plasma 25(OH) levels by dose received at 6 months and 12 months based on levels of 25(OH) at baseline. Taken from Hin et al, 2017[20]

	6 months			12 months		
Baseline 25(OH)D nmol/L	4000 IU	2000 IU	Difference	4000 IU	2000 IU	Difference
<36	115 (5.0)	89 (4.6)	26 (6.8)	123 (5.0)	91 (4.5)	32 (6.7)
36 to <48	112 (4.0)	93 (4.8)	20 (6.3)	120 (3.9)	98 (4.6)	22 (6.1)
48 to <60	132 (5.0)	92 (5.9)	40 (7.7)	141 (5.1)	103 (5.9)	38 (7.8)
60+	145 (4.9)	111 (4.0)	34 (6.3)	167 (5.6)	116 (4.6)	50 (7.3)

Arithmetic mean (SE) shown. Means and SEs are adjusted for baseline values, with missing data imputed using multiple imputation.

Four baseline groups defined by the quartiles of the distribution in all participants.

Figure S1: The association between iron stores in the form of ferritin with biochemical markers for iron release (hepcidin), transferrin (Tf), Tf saturation (TSAT) and red cell output from the bone marrow (sTfR and sTfR-ferritin index). The line of best fit using simple linear regression is shown for unadjusted baseline data.

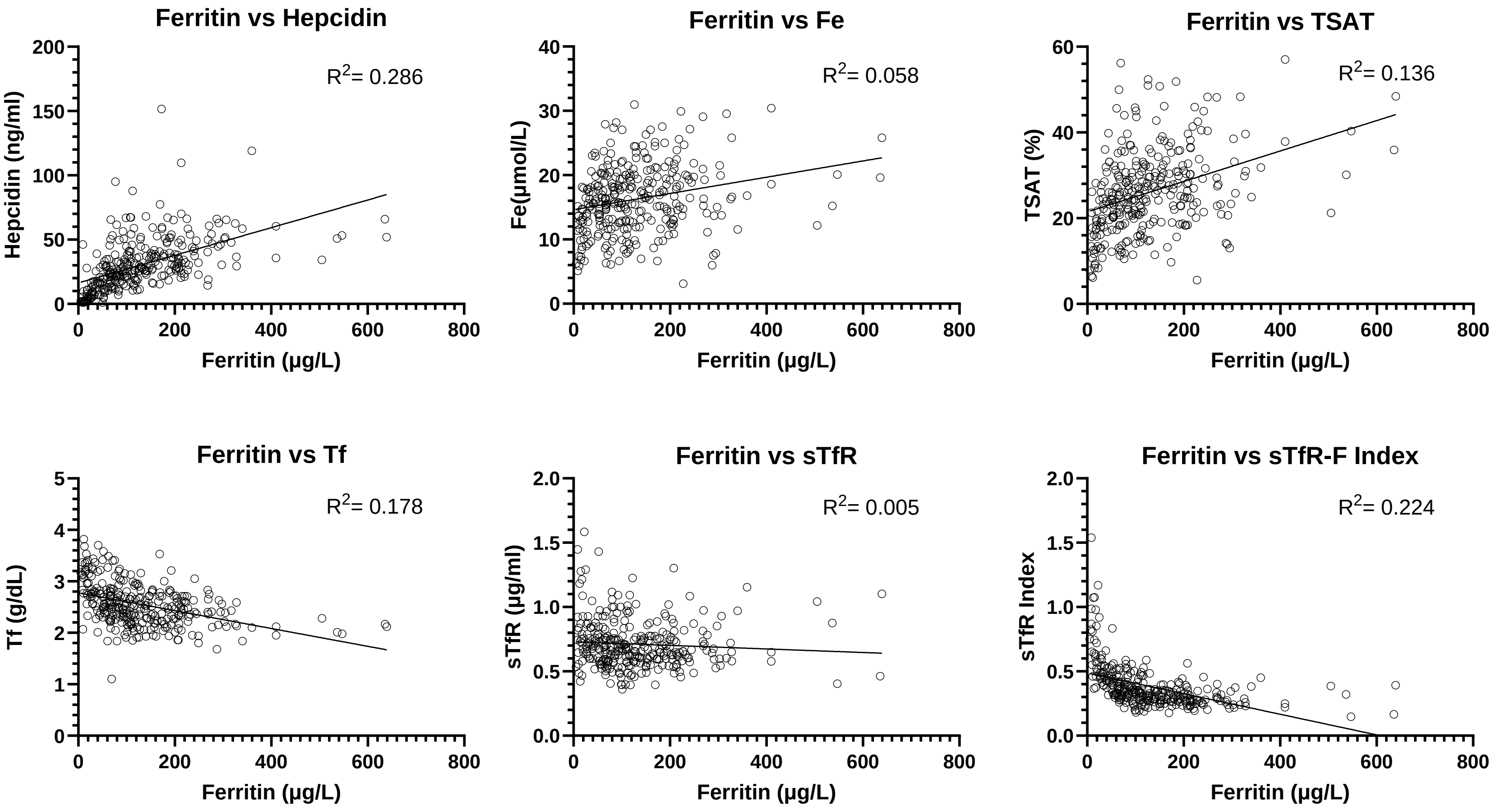

Table S3: Correlations between baseline measures of iron status

	Ln hepcidin	Ln sTfR	Ln ferritin	Iron	Transferrin	TSAT	Ln sTfR:Ferritin index
Ln hepcidin	1	-0.24	0.78	0.2	-0.54	0.33	-0.65
Ln sTfR	.	1	-0.14	-0.24	0.13	-0.26	0.8
Ln ferritin	.	.	1	0.29	-0.51	0.41	-0.71
Iron	.	.	.	1	-0.07	0.89	-0.36
Transferrin	.	.	.	.	1	-0.48	0.4
TSAT	.	.	.	.	.	1	-0.44
Ln sTFR:Ferritin index	.	.	.	.	.	.	1

Pearson (partial) correlation coefficients shown, adjusted for age and sex.

Table S4a: Post-hoc assessment of the effect of allocation to vitamin D3 4000 IU or 2000 IU daily versus placebo on 12-month concentrations of iron biomarkers subdivided by baseline 25(OH)D concentration (below/above median).

	**Either dose (n=204)**	**Placebo (n=101)**	**Difference**	**P**
Ln hepcidin, ln ng/mL				
Baseline 25(OH)D below median	2.97 (0.069)	2.99 (0.085)	-0.02 (0.110)	0.84
Baseline 25(OH)D above median	3.09 (0.052)	3.03 (0.081)	0.06 (0.096)	0.53
Ln sTfR, ln μg/mL				
Baseline 25(OH)D below median	-0.36 (0.022)	-0.36 (0.029)	-0.00 (0.037)	0.98
Baseline 25(OH)D above median	-0.38 (0.018)	-0.36 (0.030)	-0.01 (0.035)	0.68
Ln ferritin, ln μg/L				
Baseline 25(OH)D below median	4.49 (0.048)	4.56 (0.060)	-0.07 (0.077)	0.33
Baseline 25(OH)D above median	4.66 (0.034)	4.60 (0.057)	0.06 (0.066)	0.34
Iron, umol/L				
Baseline 25(OH)D below median	15.9 (0.61)	16.6 (0.79)	-0.7 (1.00)	0.47
Baseline 25(OH)D above median	17.4 (0.45)	18.2 (0.73)	-0.8 (0.86)	0.37
Transferrin, g/dL				
Baseline 25(OH)D below median	2.6 (0.02)	2.6 (0.03)	-0.0 (0.04)	0.46
Baseline 25(OH)D above median	2.6 (0.02)	2.6 (0.03)	-0.1 (0.03)	0.12
TSAT, %				
Baseline 25(OH)D below median	25.1 (0.92)	26.6 (1.18)	-1.5 (1.50)	0.32
Baseline 25(OH)D above median	27.8 (0.77)	28.7 (1.21)	-0.8 (1.43)	0.57
Ln sTFR:Ferritin index				
Baseline 25(OH)D below median	-0.99 (0.027)	-1.02 (0.035)	0.03 (0.044)	0.45
Baseline 25(OH)D above median	-1.07 (0.020)	-1.04 (0.033)	-0.02 (0.039)	0.52

Mean (SE) shown. Estimates are adjusted for the baseline values. Missing data are imputed using multiple imputation.

Table S4b: Post-hoc assessment of the effect of allocation to vitamin D3 4000 IU or 2000 IU daily versus placebo on 12-month concentrations of iron biomarkers subdivided by baseline 25(OH)D concentration (below/above 30 nmol/L).

	**Either dose (n=204)**	**Placebo (n=101)**	**Difference**	**P**
Ln hepcidin, ln ng/mL				
Baseline 25(OH)D <30 nmol/L	3.04 (0.159)	3.09 (0.210)	-0.05 (0.263)	0.86
Baseline 25(OH)D ≥30 nmol/L	3.03 (0.046)	3.01 (0.062)	0.02 (0.077)	0.84
Ln sTfR, ln μg/mL				
Baseline 25(OH)D <30 nmol/L	-0.41 (0.043)	-0.42 (0.057)	0.01 (0.071)	0.84
Baseline 25(OH)D ≥30 nmol/L	-0.37 (0.015)	-0.35 (0.022)	-0.01 (0.027)	0.59
Ln ferritin, ln μg/L				
Baseline 25(OH)D <30 nmol/L	4.26 (0.121)	4.72 (0.161)	-0.46 (0.201)	0.0323
Baseline 25(OH)D ≥30 nmol/L	4.61 (0.029)	4.57 (0.043)	0.04 (0.052)	0.44
Iron, umol/L				
Baseline 25(OH)D <30 nmol/L	14.8 (0.99)	15.5 (1.33)	-0.7 (1.65)	0.68
Baseline 25(OH)D ≥30 nmol/L	16.9 (0.41)	17.5 (0.59)	-0.6 (0.71)	0.42
Transferrin, g/dL				
Baseline 25(OH)D <30 nmol/L	2.6 (0.05)	2.5 (0.07)	0.1 (0.09)	0.19
Baseline 25(OH)D ≥30 nmol/L	2.6 (0.01)	2.6 (0.02)	-0.1 (0.03)	0.0404
TSAT, %				
Baseline 25(OH)D <30 nmol/L	23.4 (1.63)	26.2 (2.20)	-2.8 (2.74)	0.31
Baseline 25(OH)D ≥30 nmol/L	26.9 (0.64)	27.6 (0.92)	-0.8 (1.12)	0.50
Ln sTFR:Ferritin index				
Baseline 25(OH)D <30 nmol/L	-0.99 (0.062)	-1.11 (0.082)	0.12 (0.103)	0.25
Baseline 25(OH)D ≥30 nmol/L	-1.04 (0.017)	-1.02 (0.025)	-0.01 (0.030)	0.64

Mean (SE) shown. Estimates are adjusted for the baseline values. Missing data are imputed using multiple imputation.
